# Cancer cachexia: the elephant in the room?

**DOI:** 10.1002/jcsm.12911

**Published:** 2021-12-27

**Authors:** Eric J. Roeland

**Affiliations:** ^1^ Knight Cancer Institute Oregon Health and Science University Portland OR USA

When confronting new and complex challenges, I often think of the parable of the blind men and the elephant. In meeting an elephant for the first time, each blind man touches a different part of the elephant and describes it based on his limited experience. While each one's first‐hand account is true, it's not the totality of the truth. This parable illustrates the limits of perception and the importance of understanding the full context. Moreover, it highlights the importance of combining our perspectives for a complete view.

Cancer cachexia is our elephant. Despite its high prevalence and ubiquitous impact on patients with cancer, we still lack a clear, clinically meaningful, and universally accepted definition. We have a widely cited international consensus working definition,^1^ and we eagerly anticipate its update underway; however, evidence supporting a definition harmonizing its complex pathophysiology and diverse clinical impact is lacking. We each describe cachexia based on our own clinical experience and/or research vantage points, including weight, skeletal muscle, physical function, food intake, metabolism, inflammation, treatment intensity, quality of life, healthcare utilization, and survival. Contrasting criteria for cancer cachexia continue to undermine research and clinical practice and, most notably, the approval of drugs by regulatory agencies. Consequently, defining cancer cachexia remains a central challenge within our field.

In this issue,^2^ Martin *et al*. present the first international multicentre study focused on three critical aspects of cancer cachexia—linking the association between reduced food intake, cancer‐associated weight loss, and survival. Without existing standards for measuring cancer cachexia, I applaud their efforts to coalesce a large, international dataset of more than 12 000 patients with cancer. Building upon prior efforts to characterize cancer‐associated weight loss,^3^ these findings suggest that reduced food intake predicts a high likelihood of severe weight loss. Although these data may seem obvious to most practicing oncologists, it is startling that contemporary large‐scale datasets such as these to describe cancer cachexia are lacking. As we place these findings within the preclinical and clinical context, I believe we should reflect on three key themes: (i) sharing data, (ii) predicting clinical outcomes, and (iii) trusting our patients' own words.

## Sharing data

Cachexia data are largely unavailable. Surprisingly, cancer cachexia is not included in national cancer statistics in any country, and there exist no large data repositories. Determined investigators have compiled retrospective data, but this information is sparse and at high risk of sampling bias. We require consensus by acquiring large, contemporary representative datasets to validate a clinically meaningful definition. A comprehensive dataset is only possible through an international collaboration of research teams, institutions, and nationally sponsored cooperative groups. Ideally, by acquiring extensive representative data, we can standardize definitive cachexia criteria and include essential updates towards cancer cachexia's inclusion within the International Classification of Diseases (ICD). Aligned with this aim, I believe that Martin *et al*. demonstrate how the combination and analysis of prospectively collected data amassed across distinct institutional datasets can promote the extraction of real‐world data and promote predictive modeling.

## Predicting clinical outcomes

What are the biological and clinical predictors of cancer cachexia? This team of investigators utilized multinomial logistic regression, a simple way to determine how we can explain the variance in weight loss by the different hypothesized factors. This analytic approach has clear limitations, but it does allow us to gauge the relative effect sizes of the different covariables, including tumor type, stage, sex, age, performance status, food intake, and biological indices (e.g. inflammation) as well as the extent of unexplained existing residual variation. Herein, we realize the enormous effect of food intake as captured by three simple, patient‐reported categories; conversely, we observe the relatively small (and surprising) impact of C‐reactive protein. As we continue to unravel the complex biological mechanisms of cachexia, some may identify inflammation (e.g. IL‐1, IL‐6, and TNF) and nefarious tumour molecules [e.g. growth differentiation factor 15 (GDF‐15)^4^] as the critical drivers of cachexia. Others contend that low food intake is largely the culprit. Both may be right insofar as inflammatory mediators may suppress appetite through central mechanisms to elicit catabolism, rather than primarily acting on skeletal muscle and/or adipose tissue.

## Trusting our patients' own words

As a practicing oncologist, I listen to and trust my patients' ability to express their unique and personal experiences with cancer. Patient‐reported outcomes (PROs) have become a science. Longitudinal changes in PROs shift the focus away from the tumor and towards the patient. In fact, we routinely measure complex biology and clinical symptoms with PROs, including pain, dyspnea, fatigue, and nausea/vomiting. Sometimes the most complex pathophysiology is best summarized by patients using their own words. Here, Martin *et al*. utilize validated PROs to measure food intake (i.e. PG‐SGA‐SF^5^ and Ingesta Score^6^) and apply methods to unify these measures. These assessments are clinically practical to identify patients at risk and actionable to target patients with low food intake to optimize their symptom management and nutrition. Based on its association with weight loss, this analysis provides a simple criterion for reduced food intake that investigators can prospectively validate.

All aspects of cancer and cancer cachexia are incredibly complex. As we gain additional insights into cancer cachexia's intricate pathophysiology and develop novel treatments, we also glean key insights into specific patient factors impacting cancer treatment delivery, the risk for toxicity, quality of life, and survival. We can simultaneously treat cancer *and* the patient. As a solution‐focused community, we must share data, predict clinical outcomes, and trust our patients to define this elephant in the room.

## Conflict of interest

E.J.R. has served as a consultant for Asahi Kasei Pharmaceuticals, DRG Consulting, Napo Pharmaceuticals, and American Imaging Management. Additionally, he has served on recent advisory boards for Heron Pharmaceuticals, Pfizer, Vector Oncology, and Helsinn Pharmaceuticals. He has also served as a member on data safety monitoring boards for Oragenics, Inc., Galera Pharmaceuticals, and Enzychem Lifesciences Pharmaceutical Company.
